# Genetically Similar High-Risk Strains of Carbapenemase-Producing Enterobacterales in Humans and Companion Animals, United States

**DOI:** 10.3201/eid3203.251458

**Published:** 2026-03

**Authors:** Lingzi Xiaoli, Allison E. James, Anna L. Stahl, Maho Okumura, Stephen D. Cole, Jaclyn M. Dietrich, Molly M. Leeper, Jordan K. Putney, Maroya Spalding Walters, Richard A. Stanton

**Affiliations:** Centers for Disease Control and Prevention, Atlanta, Georgia, USA (L. Xiaoli, A.E. James, A.L. Stahl, M.M. Leeper, J.K. Putney, M.S. Walters, R.A. Stanton); University of Pennsylvania School of Veterinary Medicine, Philadelphia, Pennsylvania, USA (M. Okumura, S.D. Cole, J.M. Dietrich); Applied Science Research and Technology, Inc., Smyrna, Georgia, USA (J.K. Putney); US Public Health Service, Rockville, Maryland, USA (M.S. Walters)

**Keywords:** antimicrobial resistance, bacteria, carbapenem-resistant Enterobacteriaceae, zoonoses, bacterial zoonoses, healthcare-associated infections, United States

## Abstract

To elucidate the zoonotic potential of carbapenemase-producing carbapenem-resistant Enterobacterales (CP-CRE) in US companion animals (i.e., dogs and cats), we queried the National Center for Biotechnology Pathogen Detection database to identify One Health clusters containing CP-CRE isolates from companion animals and humans. The 11 One Health clusters we found included most (69% [169/246]) publicly available CP-CRE sequences from US companion animals and were from 8 internationally disseminated, high-risk sequence types from 3 bacterial species (*Escherichia coli*, *Klebsiella pneumoniae*, and *Enterobacter cloacae*). All clustered isolates had New Delhi metallo-β-lactamase–family carbapenemases, and most (92%) carried the *bla*_NDM-5_ allele. The One Health clusters included several closely related subclusters with geographically linked isolates from both humans and companion animals. Those results suggest that CP-CRE is an emerging One Health issue and that direct or indirect transmission of CP-CRE is occurring between humans and companion animals in the United States.

Carbapenem-resistant Enterobacterales (CRE) are among the highest priority antimicrobial-resistant pathogen threats to public health in the United States and globally (*1*,*2*). Defined by resistance to the “last resort” carbapenem antibiotics, CRE infections are difficult to treat and associated with high mortality (*3*). CRE is a major cause of human healthcare-associated infections and have recently emerged as a clinical, and potentially zoonotic, pathogen in companion animals (i.e., dogs and cats) (*4*).

Enterobacterales are a taxonomic order of gram-negative bacteria that include commensal and pathogenic gastrointestinal tract organisms, such as *Escherichia coli*, *Klebsiella pneumoniae*, and *Enterobacter* spp. Carbapenem resistance in Enterobacterales species can be conferred by several different mechanisms; among those, acquisition of genes that encode carbapenemases (enzymes that inactivate carbapenems and other β-lactam antibiotics) represents the most serious public health threat (*5*). Because carbapenemase genes are often located on mobile genetic elements, they can spread rapidly through both horizontal transfer and clonal expansion (*6*,*7*). The 5 most common and widely disseminated carbapenemase families are *K. pneumoniae* carbapenemase (KPC), imipenemase metallo-β-lactamase, New Delhi metallo-β-lactamase (NDM), Verona integron-encoded metallo-β-lactamase, and oxacillinase (OXA) 48–like (*8*,*9*).

CRE isolates with carbapenemases from each of the 5 major families have been recovered from companion animals across the globe (*10*). In the United States, the earliest reported carbapenemase-producing CRE (CP-CRE) detections from companion animals were NDM-producing *E. coli* isolates collected during 2008–2009 (*11*). CP-CRE from several bacterial species and carbapenemase families have since been isolated from dogs and cats in multiple states (*12*–*18*). Although the prevalence of CP-CRE colonization (i.e., asymptomatic carriage in the gastrointestinal tract) in US companion animals was recently estimated to be only 0.2% (*16*), NDM-producing *E. coli* has caused several large outbreaks among dogs and cats in veterinary hospitals and animal rescue facilities beginning in 2018 (*14*,*17*,*18*).

Transmission of CP-CRE between humans and animals has been documented only a few times, 1 time in a household in Finland (*19*) and 2 times in veterinary hospitals in Europe and the United States (*20*,*21*). However, CP-CRE outbreaks in companion animals have included strains associated with outbreaks in human healthcare facilities, highlighting the potential for transmission between human and animal hosts (*13*,*14*,*16*,*22*). Thanks to the widespread adoption of whole-genome sequencing (WGS) for research, surveillance, and outbreak response, thousands of CP-CRE sequences from human and nonhuman sources are now publicly available. We leveraged those data to analyze the relatedness of strains circulating between humans and animals to elucidate the zoonotic potential of CP-CRE in companion animals in the United States. This activity was reviewed by the Centers for Disease Control and Prevention (CDC), deemed research not involving human subjects, and was conducted consistent with applicable federal law and CDC policy (see e.g., 45 C.F.R. part 46; 21 C.F.R. part 56; 42 U.S.C. §241(d), 5 U.S.C. §552a, 44 U.S.C. §3501 et seq.).

## Materials and Methods

### Companion Animal Isolate and One Health Cluster Identification

**Figure 1:**
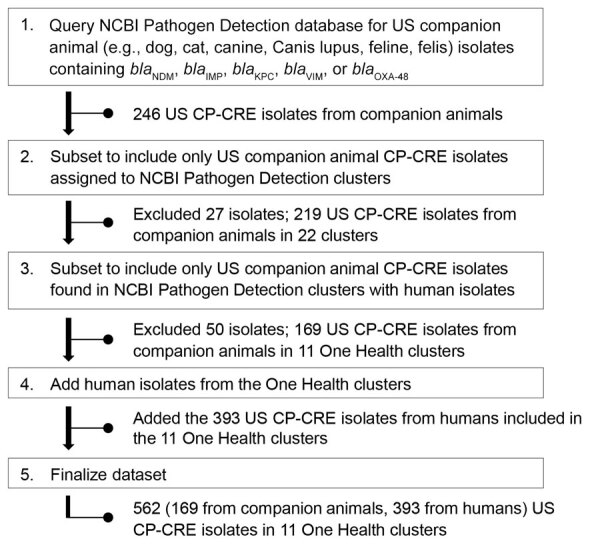
Workflow for identification and inclusion of genetically related US companion animal and human CP-CRE isolates using the NCBI Pathogen Detection database used in study of genetically similar high-risk strains of carbapenemase-producing Enterobacterales in humans and companion animals, United States. CP-CRE, carbapenemase-producing carbapenem-resistant Enterobacterales; IMP, imipenemase metallo-β-lactamase; KPC, K. pneumoniae carbapenemase; NCBI, National Center for Biotechnology Information; NDM, New Delhi metallo-β-lactamase; OXA, oxacillinase; VIM, Verona integron-encoded metallo-β-lactamase.

We queried the National Center for Biotechnology Information (NCBI) Pathogen Detection database (https://www.ncbi.nlm.nih.gov/pathogens) on October 24, 2023, to identify Enterobacterales isolates from US dogs and cats containing any of the 5 major carbapenemase gene families (*23*) (Figure 1). We designated Pathogen Detection clusters (predefined by NCBI as isolates within <25 allele differences from species-specific, whole-genome multilocus sequence typing schemes) containing CP-CRE collected from companion animal and human sources from the United States as One Health clusters for further analysis. We continued to add isolates to the One Health clusters through February 23, 2024.

### Metadata and Epidemiologic Data Collection

We downloaded metadata for isolates belonging to One Health clusters from the Pathogen Detection database (including host species, location, isolation source, etc.). Additional anonymized isolate and patient characteristic data were obtained for analyses, including patient state of residence, isolation source, and specimen type (i.e., colonization or clinical test). When specimen type information was missing, rectal swab samples were categorized as colonization tests, and samples from all other body sites were considered clinical specimens. Those additional data were obtained from the CDC Antimicrobial Resistance Laboratory Network (AR Lab Network), the University of Pennsylvania Veterinary Diagnostic Laboratory, and the Microbiology Laboratory at Texas A&M University Veterinary Medical Teaching Hospital. Linked data were not available or not requested from 7 human-origin sequences and 4 animal-origin sequences; for those sequences only, we used the metadata available in Pathogen Detection. To ensure confidentiality, state of residence for both human and animal patients were classified only by their AR Lab Network region of residence (*24*).

### Bioinformatics Analysis

For each One Health cluster, we downloaded available isolate sequence assemblies from NCBI or generated with SKESA version 3.0.0 (*25*) with reads downloaded from the NCBI Sequence Read Archive (https://www.ncbi.nlm.nih.gov/sra) for samples without available assemblies. We identified the multilocus sequence types (STs) for all isolates using mlst version 2.23.0 (https://github.com/tseemann/mlst) with PubMLST typing schemes (*26*). We determined genetic similarity among CP-CRE sequences of human-origin and animal-origin isolates within the same ST by core-genome multilocus sequence typing (cgMLST) to provide a standardized basis of comparison across multiple STs and cluster sizes. We applied publicly available cgMLST schemes for *E. coli* (2,513 loci) from EnteroBase (*27*) and *K. pneumoniae* (2,537 loci) from Institut Pasteur (*28*) as previously described (*29*). For *Enterobacter cloacae*, we constructed an ad hoc cgMLST scheme with 4,229 loci from the 41 CP-CRE isolates within the identified One Health clusters using Roary (https://github.com/rastanton/cgMLST_Scripts; *30*). We constructed phylogenetic dendrograms from cgMLST allele differences using the unweighted pair group method with arithmetic mean. We annotated the cgMLST trees with AR Lab Network regions of collection and host species (dog, cat, and human) using iToL version 4.0 (https://github.com/tseemann/mlst). We calculated the cgMLST allele differences within each cluster for different host pairs (e.g., human–human, human–animal, animal–animal) and summarized them using statistics tools from NumPy (*31*).

### Data Validation

We verified isolate host information with epidemiologic data. We excluded isolate sequences if they were from sources other than humans or companion animals, they were duplicate sequences from the same isolate, if an isolate was <3 cgMLST allele differences from another isolate collected from the same patient on the same day, or if the sequence was not from paired-end reads.

## Results

### Dataset Generation

As of February 23, 2024, a total of 246 CP-CRE isolate sequences from US companion animals were available in the NCBI Pathogen Detection database (Figure 1), 26 isolates from cats (11%) and 220 isolates from dogs (89%). Most isolates harbored *bla*_NDM_ (236 [96%]). Among the isolates with *bla*_NDM_, 56% (n = 131) were *E. coli*, 31% were *E. cloacae* (n = 72), and 14% were *K. pneumoniae* (n = 33). Nine isolates harbored *bla*_KPC_; 7 were *E. cloacae*, 1 was *E. coli*, and 1 was *Klebsiella oxytoca*. A single OXA-48–like–producing *K. oxytoca* isolate from companion animals was also identified.

Among the 246 US isolates from companion animals, 169 (69%) belonged to 11 One Health clusters (Table 1; Appendix 1 Table 1), which included 393 human-origin isolates. All clustered isolates were collected during January 2016–February 2024, a period that marked a rapid increase in the use of WGS; 10 times more US CP-CRE sequences were uploaded to NCBI in 2023 than were uploaded in 2016 (Appendix 2 Figure 1).

**Table 1:** Companion animal and human CP-CRE isolates included in One Health clusters in study of genetically similar high-risk strains of carbapenemase-producing Enterobacterales in humans and companion animals, United States*

Characteristic	Companion animal	Human
No. isolates†	169	393
Specimen type	n = 168	n = 388
Colonization test	126 (75)	21 (5)
Clinical test	42 (25)	367 (95)
Source (for clinical tests)	n = 42	n = 365
Respiratory Tract	14 (33)	8 (2)
Urine	11 (26)	261 (72)
Wound	12 (29)	17 (5)
Blood	0	48 (13)
Other	5 (12)	31 (8)
No. unique patients	n = 158‡	n = 386§
Dogs	145 (92)	NA
Cats	13 (8)	NA
Patient region of residence¶	n = 154	n = 379
Central	9 (6)	32 (8)
Mid-Atlantic	78 (51)	60 (16)
Midwest	22 (14)#	41 (11)
Mountain	1 (1)	89 (23)
Northeast	41 (27)	57 (15)
Southeast	3 (2)	36 (9)
West	0	64 (17)

*Values are no. (%) except as indicated. CP-CRE, carbapenemase-producing carbapenemase-resistant Enterobacterales; NA, not applicable. †Isolates or patients with missing data were excluded from corresponding denominators. ‡Eleven unique isolates were collected from 1 cat and 6 dogs. §Seven unique isolates were collected from 5 humans. ¶Centers for Disease Control and Prevention Antimicrobial Resistance Laboratory Network regions (*24*). #Includes 18 animals known to have been imported from other countries.

### Isolate and Patient Characteristics

Among the 562 isolates in One Health CP-CRE clusters, *E. coli* was the most common species (88%, n = 493), followed by *E. cloacae* (7%, n = 41) and *K. pneumoniae* (5%, n = 28) (Table 2). All isolates harbored NDM-family carbapenemases; 92% (n = 519) had *bla*_NDM-5_ and 8% (n = 43) had *bla*_NDM-7_. Seven isolates from humans, all from the largest *E. coli* One Health cluster (ST167 cluster 3) (Table 2), also carried carbapenemases from different families (4 with OXA-48–like and 3 with KPC genes) (Appendix 1 Table 2) in addition to *bla*_NDM-5_.

**Table 2:** One Health clusters of CP-CRE isolates in study of genetically similar high-risk strains of carbapenemase-producing Enterobacterales in humans and companion animals, United States*

Species	ST	Carbapenemase gene	No. isolates			
			Human	Dog	Cat	Total
*Escherichia coli*	ST162 cluster 1	*bla*_NDM-7_	1	1	0	2
	ST162 cluster 2	*bla*_NDM-5_	2	1	0	3
	ST167 cluster 1	*bla*_NDM-5_	9	3	0	12
	ST167 cluster 2	*bla*_NDM-5_	10	55	4	69
	ST167 cluster 3*	*bla*_NDM-5_	275	10	0	285
	ST361	*bla*_NDM-5_	34	21	0	55
	ST410	*bla*_NDM-5_	27	2	0	29
	ST617	*bla*_NDM-5_	24	12	2	38
*Klebsiella pneumoniae*	ST11	*bla*_NDM-5_	1	15	2	18
	ST307	*bla*_NDM-5_	5	4	1	10
*Enterobacter cloacae*	ST171	*bla*_NDM-7_	5	31	5	41

*One human isolate belonged to ST12266. CP-CRE, carbapenemase-producing carbapenemase-resistant Enterobacterales; ST, sequence type.

Among isolates with available data, 75% (126/168) of companion animal-origin isolate sequences were collected for colonization screening, compared with 5% (21/388) of the human isolates (Table 1). Seventy-two percent of human clinical isolates were collected from urine (261/365), whereas those from companion animals were divided roughly equally among the respiratory tract (tracheal wash or bronchoalveolar lavage samples, 33%, 14/42), wounds (29%, 12/42), and urine (26%, 11/42).

The One Health cluster isolates were from 386 unique human patients and 158 companion animal patients. Compared with the broad geographic distribution of human-origin isolates, companion animal isolates were concentrated in 2 neighboring regions; 77% (119/154) were from the Mid-Atlantic or Northeast.

### Cluster Characteristics and Genetic Analyses

Of the 11 One Health clusters, 8 were from *E. coli*. The 11 clusters were composed of 8 unique STs; 2 *E. coli* STs were associated with multiple clusters (ST162 [2 clusters] and ST167 [3 clusters]) (Table 2). The size of the clusters varied from 2 isolates (*E. coli* ST162 cluster 1) to 285 isolates (*E. coli* ST167 cluster 3) (Table 2). Seven clusters contained isolates collected from dogs, cats, and humans, whereas 4 contained isolates collected from humans and dogs only. The fraction of human isolates within clusters varied from 6% (*K. pneumoniae* ST11 [1/18]) to 96% (*E. coli* ST167 cluster 3 [275/285]). Of the CP-CRE isolates from companion animals that were not part of One Health clusters, 59% (49/77) were from the same STs as the One Health clusters (*E. coli* ST162 and ST167, *K. pneumoniae* ST307, and *E. cloacae* ST171) (Appendix 1 Table 1).

**Figure 2:**
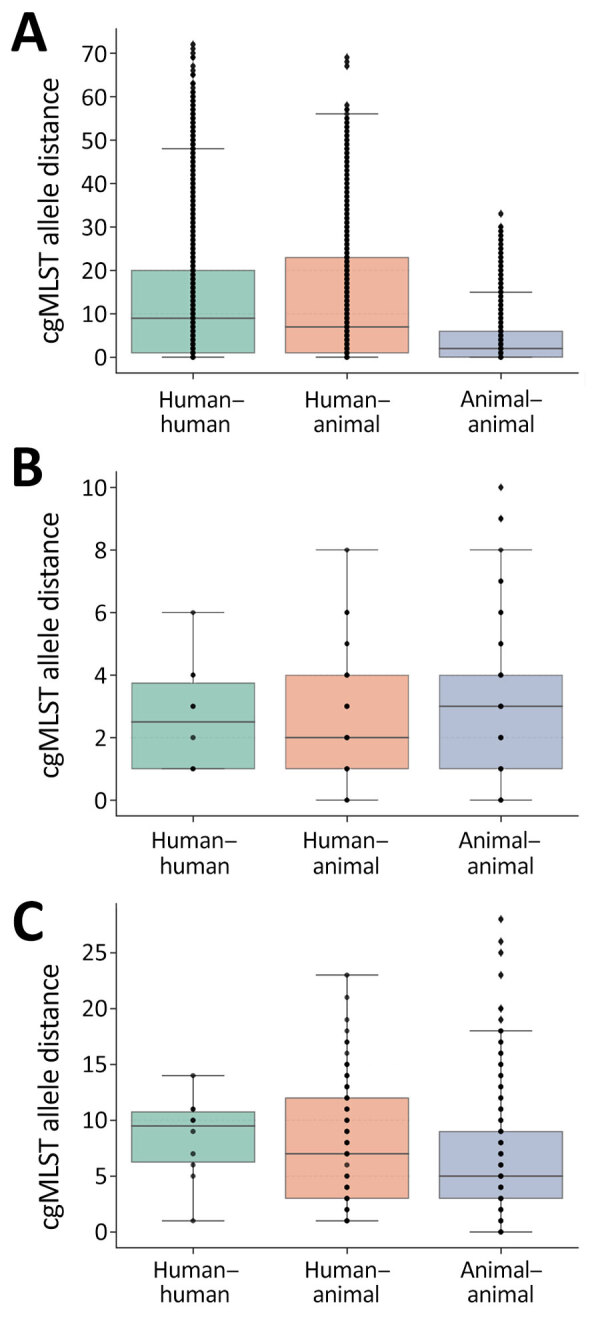
Frequency boxplots of pairwise within-cluster cgMLST allele distances among carbapenemase-producing carbapenem-resistant Enterobacterales isolates collected from humans and companion animals in study of genetically similar high-risk strains of carbapenemase-producing Enterobacterales in humans and companion animals, United States. Pairwise cgMLST allele distances were calculated between pairs within individual clusters and depicted by bacterial species with *Escherichia coli* (A), *Klebsiella pneumoniae* (B), and *Enterobacter cloacae* (C). Box top and bottom boundaries depict 25th and 75th quartiles, horizontal lines within boxes depict median values, dots represent individual data points, and whiskers represent datapoints within 1.5 times the interquartile range. cgMLST, core-genome multilocus sequence typing.

To investigate whether the isolates from humans and companion animals within One Health clusters were genetically distinct from one another, we compared cgMLST allele differences between human–human, human–animal, and animal–animal isolate pairs (examples in Figure 2). The interquartile ranges overlapped across all 3 pairwise categories. The median allele difference for human–animal pairs was lower than that for human–human pairs across all 3 CP-CRE species. Plots of the relative frequencies of within-cluster pairwise allele differences by CP-CRE species also showed overlapping human–human and human–animal pair distributions (Appendix 2 Figure 2).

**Figure 3:**
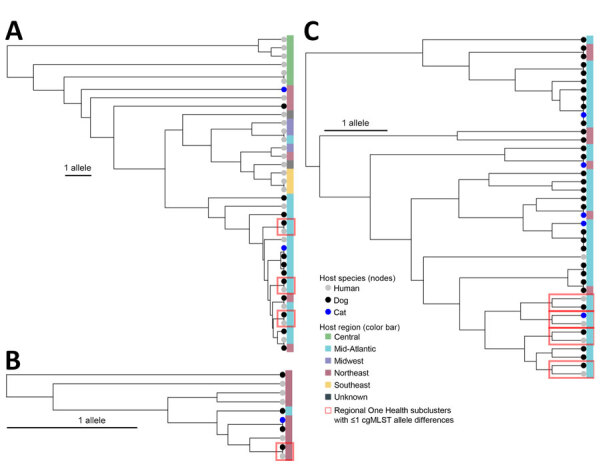
Phylogenetic core-genome multilocus sequence typing trees of *Escherichia coli* sequence type (ST) 617, *Klebsiella pneumoniae* ST307, and *Enterobacter cloacae* ST171 One Health clusters in study of genetically similar high-risk strains of carbapenemase-producing Enterobacterales in humans and companion animals, United States. The tree nodes are colored by host species, and the bands on the right are colored by the region of patient residence. cgMLST, core-genome multilocus sequence typing.

Eight of the One Health clusters (representing each of the 3 species and all STs) contained human–animal isolate pairs that were 0–1 cgMLST allele differences apart (Appendix 1 Table 2). Seven of those clusters included human–animal pairs that were related within 0–1 cgMLST allele differences and collected from the same region (Figure 3).

## Discussion

Our analysis of CP-CRE sequences in the NCBI Pathogen Detection database found >240 isolates collected from US companion animals; nearly 70% clustered with isolates from humans. Those One Health clusters included isolates from 3 different bacterial species and 8 unique STs, and all harbored NDM-family carbapenemase genes. All cluster isolates were collected during a period that coincided with the rapid emergence of the NDM family of carbapenemase genes in US human patients (*32*,*33*). The One Health clusters included very closely genetically related isolate pairs from human and companion animals and many geographically linked genetic subclusters. Those findings support that emerging CP-CRE populations carried by companion animals are not genetically distinct from those isolated from humans and that strains are likely being shared among hosts.

Each of the STs identified in this analysis have been recognized as globally disseminated, high-risk strains (i.e., known to disseminate antimicrobial resistance genes) and have previously been isolated from companion animals (*13*,*16*,*34*–*38*). *E. coli* with *bla*_NDM-5_ was the most frequent species and carbapenemase allele combination in One Health clusters. Of the 5 *E. coli* STs (ST167, ST410, ST361, and ST617) identified, 4 are also among the most common NDM-5–producing human strains worldwide and were recently linked to community associated NDM-producing CRE cases in the United States (*39*–*41*). The most frequently identified of those, NDM-5–producing *E. coli* ST167, has caused outbreaks among companion animals at a veterinary hospital and an animal rescue facility in the United States and has been implicated in transmission between humans and companion animals in Europe (*19*,*20*).

Although our results provide evidence that exchange of CP-CRE between humans and companion animals is occurring in the United States, no established thresholds of relatedness (i.e., cgMLST allele differences) can be interpreted as absolute evidence of direct or indirect transmission (e.g., by exposure to a shared contaminated environment) or directionality (i.e., whether transmission occurred from humans to animals or vice versa) in the absence of clear epidemiologic links. The data do suggest that the emergence of CP-CRE among humans and companion animals in the United States is primarily driven by clonal expansion of strains that might be better suited for community spread, instead of horizontal transfer of carbapenemase genes into otherwise unrelated strains.

Most (75%) animal CP-CRE isolates in this study were found through colonization screening; those tests are used to identify persons or animals that might be asymptomatically shedding the organisms, usually to contain outbreaks or prevent introducing CP-CRE into healthcare facilities or veterinary hospitals (*42*). That finding confirms other reports that companion animals can silently carry zoonotic CP-CRE, which might accelerate spread of such organisms in community settings (*43*,*44*). Our findings of shared strains between companion animals and humans, as well as reports of outbreaks in veterinary facilities, highlight the potential risks of transmission to other companion animals, pet owners, and veterinary staff. Although the frequency of transmission is unknown, a study in Switzerland found 2 separate instances of veterinary hospital employees colonized with the same strain that had been identified in animals in their respective veterinary hospitals (*20*). That finding reinforces the importance of adhering to routine infection prevention and control measures to prevent spread within veterinary hospitals, among animal patients, and between animal patients and veterinary staff (*14*,*18*,*20*,*37*,*45*,*46*).

The first limitation of our study is that we used a convenience sample of publicly available WGS data, which are not representative of the true burden or characteristics of CP-CRE in companion animals or humans and might be skewed by the overrepresentation of closely related sequences associated with outbreaks (e.g., the *E. coli* sequences included dozens of sequences from 2 known companion animal outbreaks). In addition, most companion animal samples were from only 2 regions, the Mid-Atlantic and Northeast, the same regions in which CP-CRE outbreaks in US veterinary hospitals have been reported (*14*,*18*). Therefore, the results might underestimate the diversity and distribution of CP-CRE in companion animals across the United States.

Our results demonstrate that CP-CRE in companion animals and humans are genetically very similar and include many diverse, high-risk sequence types commonly associated with infections and outbreaks in human healthcare settings. That finding suggests that both companion animals and humans serve as reservoirs for high-risk CP-CRE strains; community reservoirs of historically healthcare-associated pathogens have the potential to increase CP-CRE infections in otherwise healthy humans and pets. Coordinated efforts between human and animal health sectors are warranted to mitigate further spread of such highly antimicrobial-resistant bacteria.

Appendix 1 Additional information from study of genetically similar high-risk strains of carbapenemase-producing Enterobacterales in humans and companion animals, United States

Appendix 2 Additional information about genetically similar high-risk strains of carbapenemase-producing Enterobacterales in humans and companion animals, United States
