# Supplementary material for: Genetically Similar High-Risk Strains of Carbapenemase-Producing Enterobacterales in Humans and Companion Animals, United States
Source: Emerg Infect Dis. 2026 Mar;32(3):341–8. doi: 10.3201/eid3203.251458 (PMC13016009; doi:10.3201/eid3203.251458)
Supplement: Appendix 2 — Additional information about genetically similar high-risk strains of carbapenemase-producing Enterobacterales in humans and companion animals, United States [file 25-1458-Techapp-s2.pdf]

Article DOI: https://doi.org/10.3201/eid3203.251458

EID cannot ensure accessibility for supplementary materials supplied by authors. Readers who have difficulty accessing supplementary content should contact the authors for assistance.

Genetically Similar High-Risk Strains of Carbapenemase-Producing Enterobacterales in Humans and Companion Animals, United States

Appendix 2

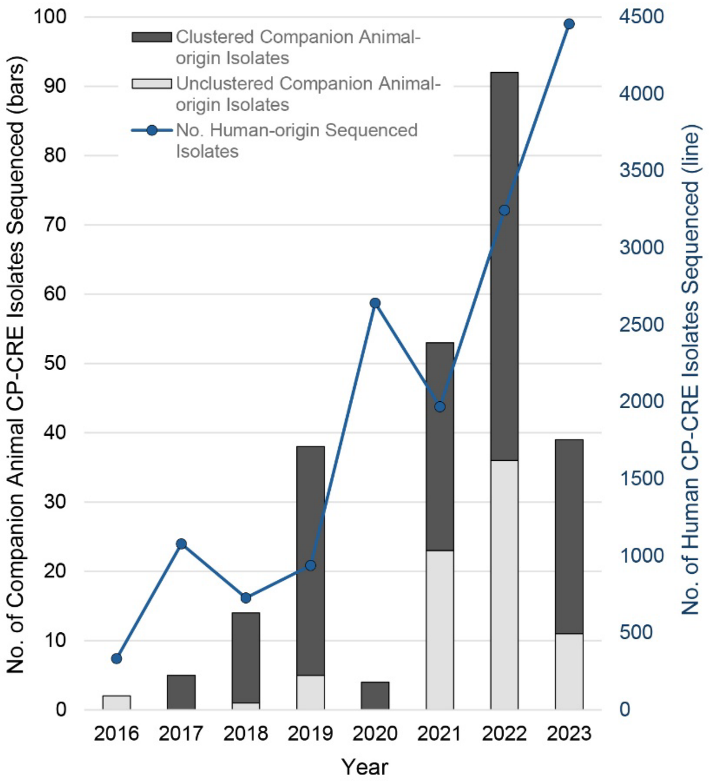

Appendix 2 Figure 1. Number of CP-CRE sequences from isolates collected from humans (blue line) and companion animals (bars) in the Pathogen Detection database during 2016–2023. Bars for companion animal-origin CP-CRE sequences are shaded to depict whether isolates belonged to One Health clusters. Data for 2024 are not shown because no companion animal sequences were uploaded in One Health clusters during January 1–February 23, 2024.

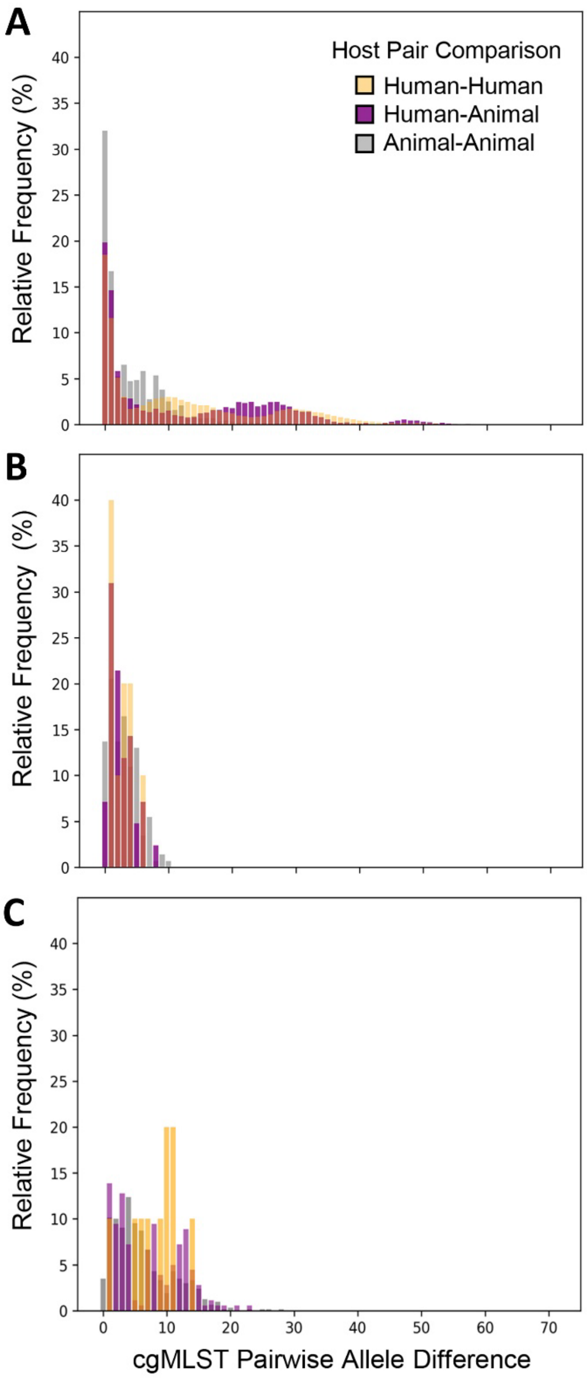

Appendix 2 Figure 2. Frequency distributions of cgMLST allele differences between human-human, human-animal, and animal-animal pairs within *E. coli*, *K. pneumoniae*, and *E. cloacae* One Health clusters. cgMLST pairwise allele differences (x-axes) are plotted relative to the frequency of each comparison (y- axes). Allele range differences were calculated within each of the eleven clusters separately but were plotted together for each bacterial species. Overlapping frequencies are depicted in red or dark yellow.

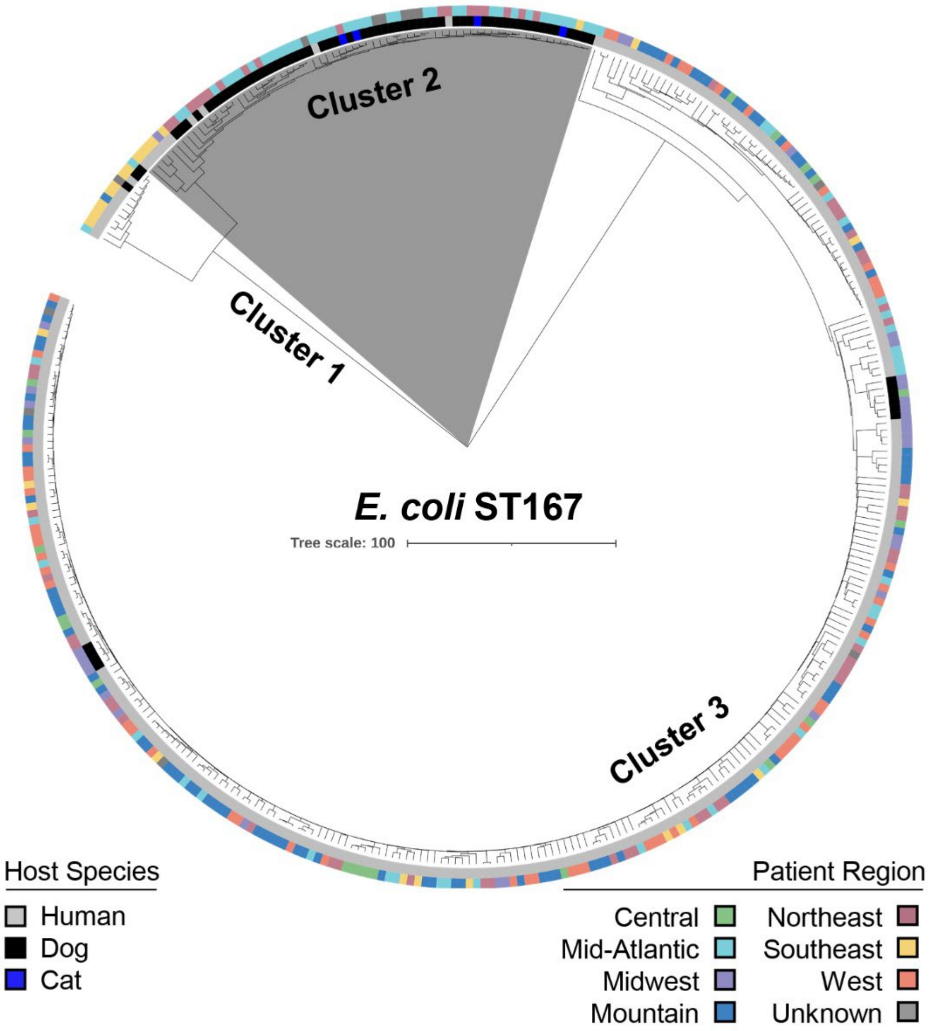

Appendix 2 Figure 3. Phylogenetic cgMLST tree of *E. coli* ST167. The inner ring depicts the host species; the outer ring shows the region of patient residence. Distinct clusters within the sequence type are labeled and depicted by shading.

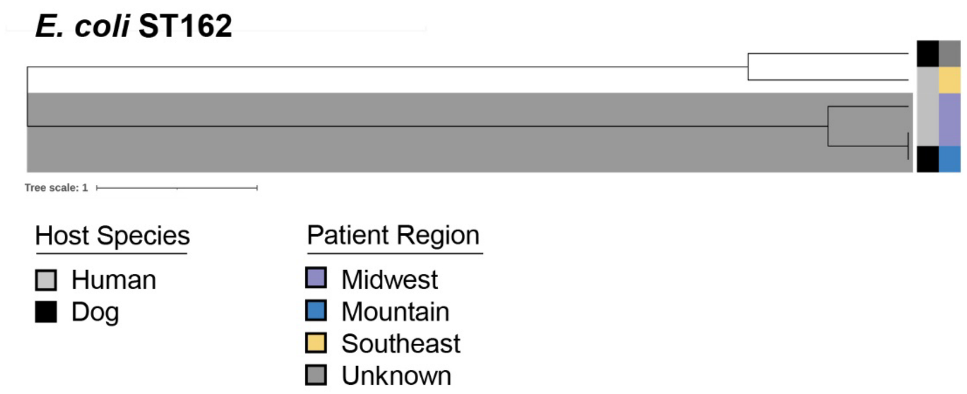

Appendix 2 Figure 4. Phylogenetic cgMLST tree of *E. coli* ST162. The left band depicts the host species; the right band shows the region of patient residence. Distinct clusters within the sequence type are labeled and depicted by shading.

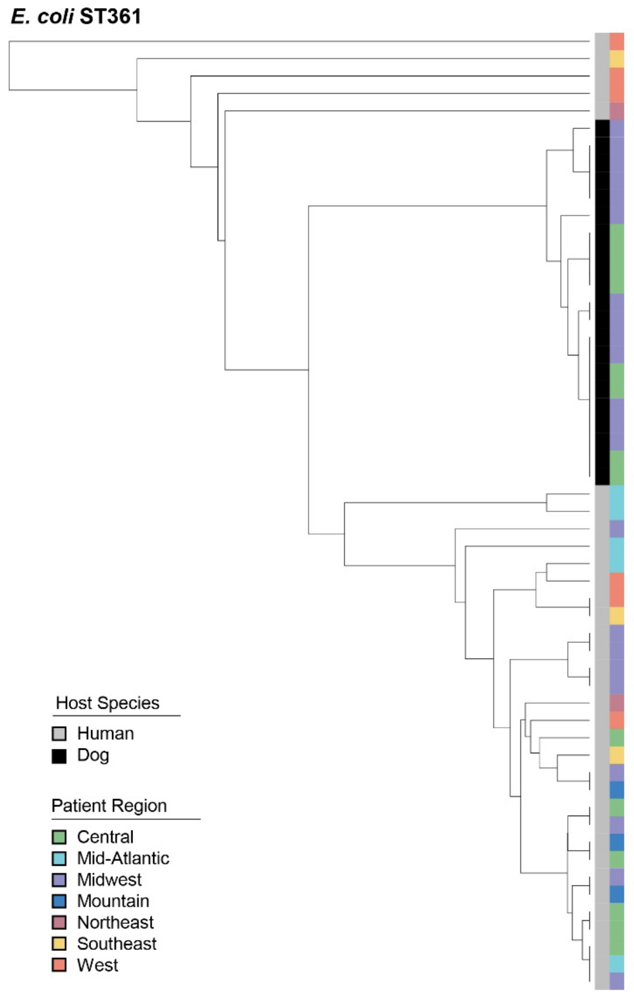

Appendix 2 Figure 5. Phylogenetic cgMLST tree of *E. coli* ST361. The left band depicts the host species; the right band shows the region of patient residence.

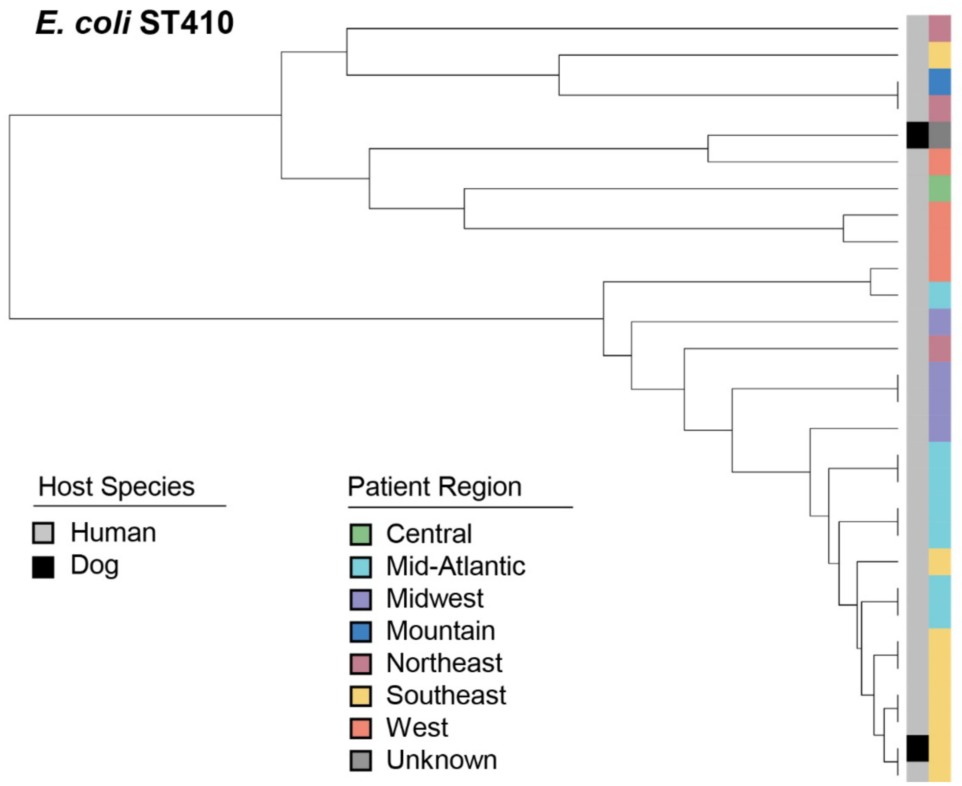

Appendix 2 Figure 6. Phylogenetic cgMLST tree of *E. coli* ST410. The left band depicts the host species; the right band shows the region of patient residence.

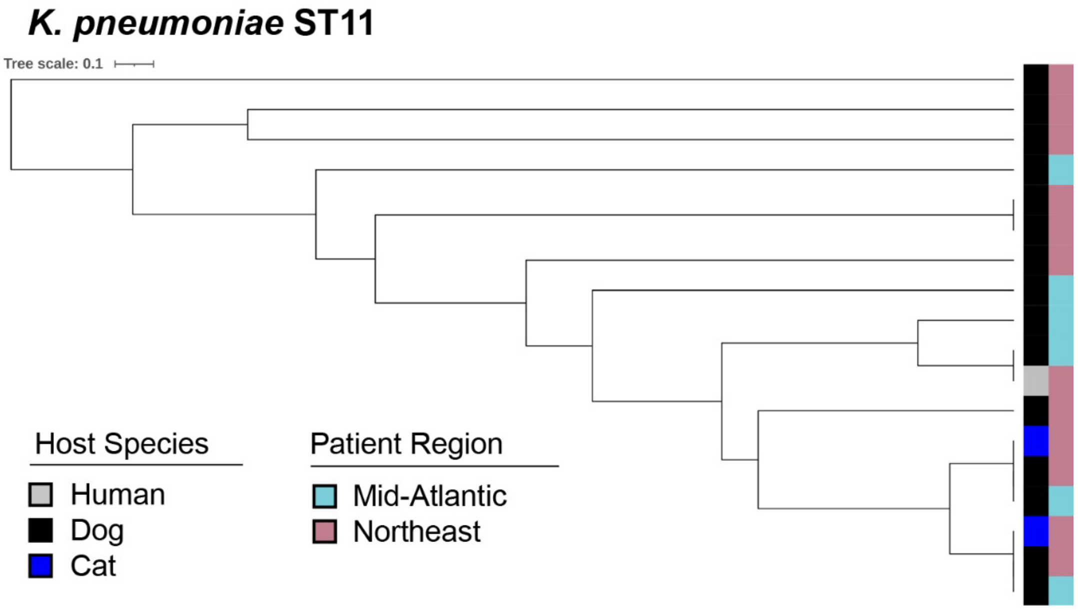

Appendix 2 Figure 7. Phylogenetic cgMLST tree of *K. pneumoniae* ST11. The left band depicts the host species; the right band shows the region of patient residence.
